# AIRE promotes androgen-independent prostate cancer by directly regulating IL-6 and modulating tumor microenvironment

**DOI:** 10.1038/s41389-018-0053-7

**Published:** 2018-05-25

**Authors:** Rashi Kalra, Ella Bhagyaraj, Drishti Tiwari, Ravikanth Nanduri, Anuja P. Chacko, Monika Jain, Sahil Mahajan, Neeraj Khatri, Pawan Gupta

**Affiliations:** Department of Molecular Biology, Council of Scientific and Industrial Research, Institute of Microbial Technology, Sector 39A, Chandigarh, 160036 India

## Abstract

Early stage prostate cancers are dependent on androgens for their growth and survival and androgen withdrawal causes them to regress. Progressive prostate cancers eventually acquire androgen independence rendering anti-androgen therapy ineffective. However, the factors leading to this have not been adequately addressed. This study shows that AIRE finds differential expression in androgen-dependent and -independent prostate cancer cells. AIRE expression is more in androgen-independent cells due to its regulation by transcription factor Elk-1. These enhanced levels of AIRE modulate the prostate tumor microenvironment by transcriptionally activating a malignancy gene IL-6 in androgen-independent cells. Additionally, AIRE prevents the cancer cells from anticancer drug-induced death and enhances their invasiveness. Moreover, AIRE by modulating the cytokine milieu skews the tumor-associated macrophage polarization towards M2 phenotype with increased CD206 and CD163 expression. Subcutaneous mouse model of prostate cancer revealed AIRE^+/+^ mice forming a palpable tumor and presents lymphadenopathy however, only a small benign tumor is observed in AIRE^−/−^ mice and lymph nodes appear normal in size. In conclusion, our findings suggest AIRE as a probable factor in promoting prostate cancer progression.

## Introduction

Prostate cancer (PCa) is the most commonly diagnosed non-cutaneous neoplasm and ranks second in cancer-related deaths among men. Despite recent advancements in the treatment of the disease, patients with the malignant disease have a poor survival rate^1^. PCa is initially androgen-dependent for its progression and androgen ablation remains to be the mainstay of therapy for patients with advanced cancers. Although this hormone withdrawal is palliative in more than 50% of the patients yet the effects are transient. However, succession to incurable androgen-independent stage where it becomes metastatic occurs within a few years in the majority of these patients^2^. Exact molecular mechanisms contributing to androgen independence are unknown, yet recent facts have highlighted the role of tumor microenvironment along with changes in androgen receptor (AR)-related functions^3–6^.

Reciprocal interactions between fibroblasts, cancer cells, and inflammatory cells release cytokines, growth factors, and angiogenic factors, which contribute to a wide variety of effects inhibiting or promoting cancer cell proliferation apart from androgen signaling axis^7,8^. Conversely, deregulated transcription factors are responsible for maintaining malignancy and demand prominence as good targets for cancer therapy^9^. AR by binding to biologically active androgens transcriptionally regulates expression of its target genes. Recently, immune gene encoding autoimmune regulator (AIRE) protein which confers autoimmune protection has been found to be regulated by androgen/AR complex in androgen-dependent LNCaP cells which endogenously express AR. Androgen recruits AR to the AIRE promoter and thereby enhances its transcription^10^. However, regulation of AIRE in androgen-independent cells comparative to the androgen-dependent cells was not investigated. AIRE is also known to be regulated by estrogen which induces epigenetic changes by increasing CpG methylated islands in the AIRE promoter and downregulates AIRE expression^11^.

Notably, AIRE in a sex steroid-dependent manner mediates gender difference in prevalence of autoimmune disorders^10,11^. AIRE’s role in preventing effective antitumor immune response has recently been clarified. AIRE-deficient mice show reduced melanoma growth due to enhanced immune rejection and increased survival^12^. Recent reports have shown that AIRE contributes to relapse-free survival in estrogen-positive breast cancer cells^13^. Given the correlation that AIRE in a hormone-dependent manner regulates the disease state; the association of AIRE in the case of other sex hormone-related cancers have not been unveiled yet. Intriguingly, we wanted to study AIRE’s role in another such cancer, i.e. prostate cancer as to how it modulates the tumor microenvironment in prostate cancer?

In this study, we report that AIRE shows a differential expression in androgen-sensitive LNCaP and androgen-insensitive PC3 cells. AIRE expression is more in PC3 cells as compared to LNCaP cells. This difference in expression is probably due to its regulation by transcription factor Elk-1 in PC3 cells. Considering this discrepancy and delving into why prostate cancer becomes androgen-independent during advanced stages, we have chosen PC3 as a cell model in major part of our study as it is the bone metastasis of grade IV prostate cancer which is untreatable. Interleukin-6 (IL-6) which is known to augment cancer cell proliferation is directly regulated by AIRE at the genomic level and rescues the drug-induced cell death in PC3 prostate cancer cells. Further, AIRE induced IL-6 and PGE2 switches the monocyte polarization to M2 phenotype. AIRE by inducing malignancy factor IL-6 leads to more of inflammation and lymphadenopathy in AIRE^+/+^ mice; however, a small benign tumor is observed in AIRE^−/−^ mice.

## Results

### Expression profiling of cytokines in the tumor microenvironment

**Fig. 1:**
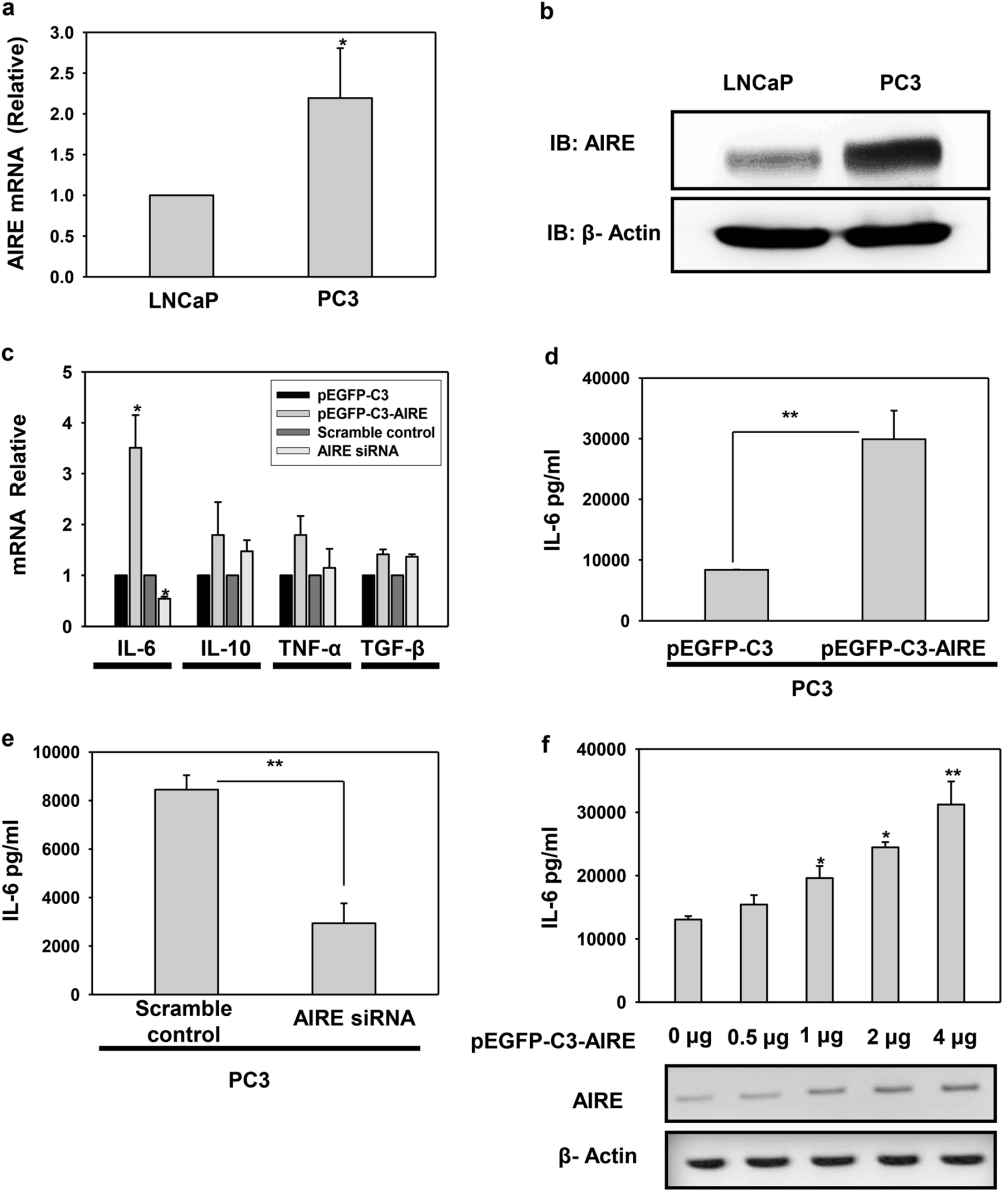
AIRE influences the expression of cytokines in the tumor microenvironment. **a** AIRE expression analysis in LNCaP and PC3 cells by qRT-PCR and **b** immunoblotting. **c** Analysis of cytokines IL-6, IL-10, TNF-α, and TGF-β in PC3 cells ectopically expressed with AIRE and in knockdown background of AIRE by qRT-PCR. **d** Levels of IL-6 secretion were assessed by ELISA in PC3 cells ectopically expressed with AIRE or **e** in the knockdown background of AIRE. **f** ELISA to measure secreted IL-6 in a dose-dependent manner of AIRE expression. Asterisks represent significant differences (*p < 0.05, **p < 0.005). Data are representative (**b**) and average (**a, c–f**) of at least three independent biological experiments performed in triplicate (mean ± s.d.)

Although AIRE is predominantly expressed in thymus, extrathymic expression of AIRE in both myeloid and epithelial lineage has been recognized since the gene has been cloned^14^. Studying AIRE in prostate cancer led us to question which cell culture model can be utilized for the study. PCa research field widely uses PC3, LNCaP, VCaP, and DU145 prostate cancer cell lines which have been derived from various metastatic lesions in prostate cancer patients^15^. To dissect the mechanism behind androgen-dependent and -independent prostate cancer we used two cell lines which are androgen sensitive, i.e. LNCaP and VCaP and two cell lines which are insensitive to androgens, i.e. PC3 and DU145 and all of them are epithelial in origin. Expression analysis of AIRE showed differential pattern; PC3 cells were expressing more AIRE as compared to LNCaP cells. This expression level was confirmed by quantitative reverse transcriptase polymerase chain reaction (qRT-PCR) and immunoblotting (Fig. 1a, b). Similar expression pattern was observed in other two androgen-dependent and -independent cell lines VCaP and DU145, respectively (Supplementary Fig. 1c, d). This discrete pattern of AIRE expression suggested that it might be playing a role in androgen-independent prostate cancer progression. Therefore, for further analysis, we used PC3 cells as a model system which is a suitable target for prostate cancer therapeutics. The conditional balance of pro-inflammatory and anti-inflammatory cytokines in the tumor microenvironment determines the antitumoral immune responses or cell transformation and malignancy^16,17^. Firstly, we profiled the transcript levels of major tumor microenvironment cytokines IL-6, IL-10, tumor necrosis factor-α (TNF-α), and transforming growth factor-β (TGF-β) in PC3 cells in both conditions: with ectopically expressed AIRE and AIRE knockdown. Complete analysis revealed the differential expression in cytokine transcript levels with a significant increase in levels of IL-6 in AIRE-overexpressed cells and notable decrease in the siRNA-mediated knockdown background of AIRE in comparison to other cytokines (Fig. 1c). AIRE overexpression and knockdown were confirmed by immunoblotting and qRT-PCR (Supplementary Fig. 1a, b). We further analyzed IL-6 expression levels by enzyme-linked immunosorbent assay (ELISA) in supernatant of cells with AIRE overexpression and knockdown and we clearly observed a significant increase in IL-6 secretion by AIRE which gets decreased in RNAi-mediated knockdown of AIRE (Fig. 1d, e). We next did a dose-dependent assay with pEGFP-C3-AIRE transfection to check its effect on IL-6 levels and we observed AIRE is regulating IL-6 secretion in a dose-dependent manner (Fig. 1f). High serum levels of IL-6 have been reported in both benign and malignant prostate cancer patients. Moreover, IL-6 is linked to the aggressive phenotype of prostate cancer by regulating the epithelial–mesenchymal transition (EMT) and bone metastasis^18,19^. This suggests the possible role of AIRE in malignancy. To look for AIRE’s general effect on IL-6 increased expression in androgen-dependent cells also, we picked up LNCaP cells for analysis and as anticipated a marginal non-significant increase was observed in cells transfected with pEGFP-C3-AIRE (Supplementary Fig. 2a).

### Elk-1 regulates AIRE expression in androgen-independent cancer cells

**Fig. 2:**
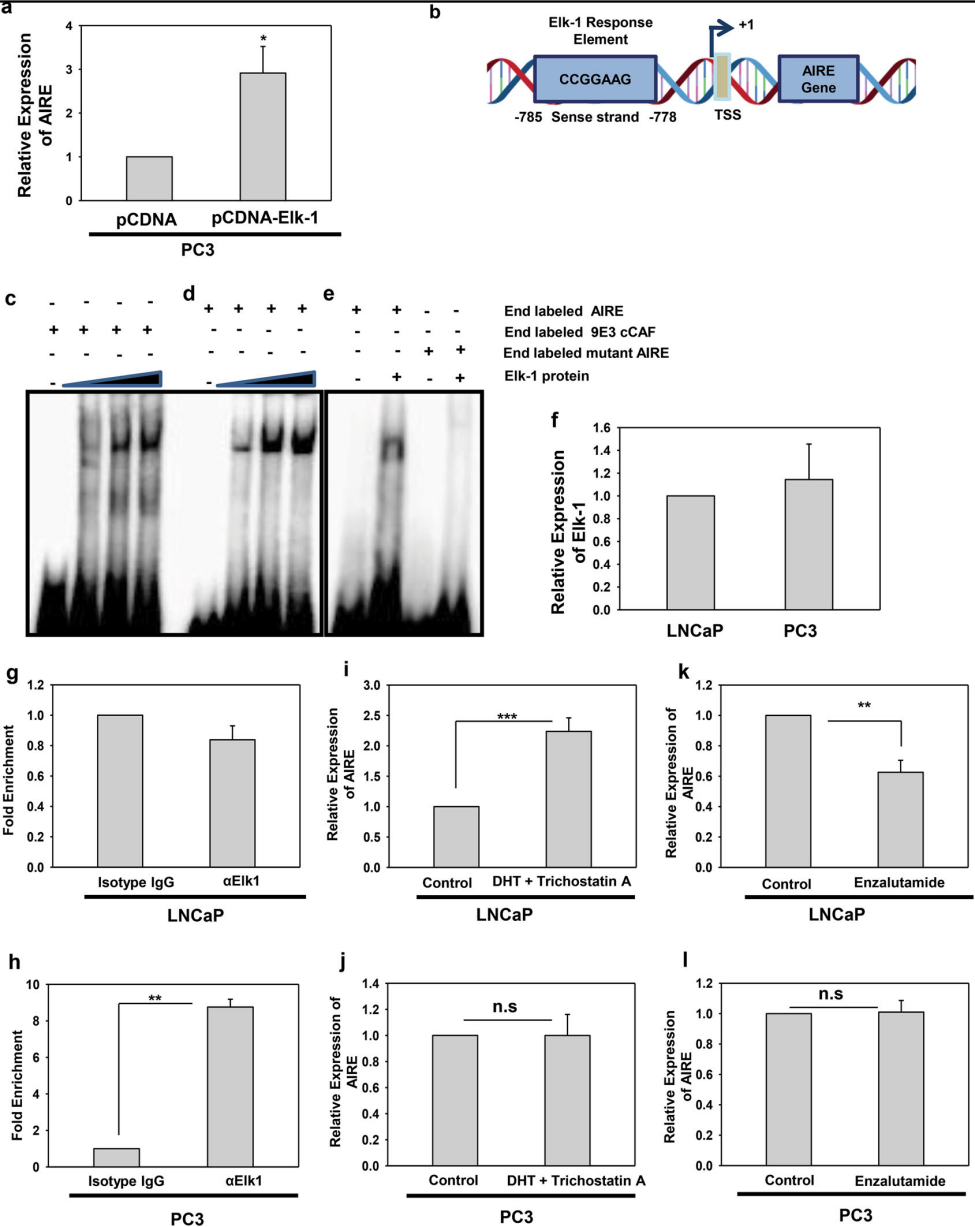
Elk-1 regulates AIRE in PC3 cells and not in LNCaP cells. **a** Expression of AIRE in PC3 cells ectopically expressed with Elk-1. **b** Pictorial representation of AIRE promoter having a binding motif for Elk-1. **c** EMSA analysis using radiolabeled oligonucleotide containing the sequence for Elk-1 binding on 9E3 cCAF regulatory region and **d** AIRE regulatory region in the presence of increasing concentration of in vitro-translated Elk-1. **e** EMSA was performed using radiolabeled oligonucleotide harboring sequence for the wild-type AIRE regulatory region and mutant in which the core motif was mutated. **f** Relative expression levels of Elk-1 in LNCaP and PC3 cells by qRT-PCR. **g** ChIP analysis of Elk-1 binding at the human AIRE promoter region was performed using chromatin isolated from LNCaP cells and **h** PC3 cells. Crosslinked chromatin was immunoprecipitated with ChIP-grade Elk-1 antibody or isotype control antibody. Isolated DNA was subjected to PCR with a specific primer pair covering a region of the AIRE gene promoter. PCRs without DNA (α-IgG) or with non-immunoprecipitated input DNA were also performed. Asterisks represent significant differences (*p < 0.05, **p < 0.005, ***p < 0.0005). **i–l** Relative mRNA expression levels of AIRE in LNCaP and PC3 cells incubated with vehicle control, 10 nM dihydrotestosterone (DHT) and 5 μM trichostatin A for 6 h and 10 μM enzalutamide for 8 h by qRT-PCR. Data are representative and represent average from three independent biological experiments performed in triplicate (mean ± s.d.)

Gene expression is regulated by the orchestration of transcription factors and other proteins which work in concert to finetune the amount of RNA being produced. Differential expression of AIRE in PC3 and LNCaP cells made us wonder what leads to this discrete expression of AIRE. AIRE promoter has already been characterized and houses sites for various cis-acting regulatory elements such as AP-1, Sp1, NF-Y, Ets1, Ets2, and AR^10,20^. To delineate the mechanism behind its distinct expression in these cells we analyzed the AIRE promoter region for the binding sites of various other transcription factors. Recently, transcription factor Elk-1 which belongs to Ets family of transcription factors has been shown to be associated with prostate cancer progression^21,22^. Elk-1 is also known to be accessory and supportive for AR transcriptional signaling^23^. Firstly, we analyzed the expression levels of AIRE in PC3 and LNCaP cells ectopically expressed with Elk-1 and we found that Elk-1 overexpression significantly increases the expression of AIRE in PC3 cells (Fig. 2a), but there was no change in expression of AIRE in LNCaP cells (Supplementary Fig. 2b). As PC3 cells do not express AR but do express Elk-1. So, we predicted if Elk-1 can regulate AIRE in PC3 cells independent of AR. We extracted AIRE promoter manually from NCBI and then examined it for the presence of Elk-1-binding site and we found a putative Elk-1 site in AIRE promoter (Fig. 2b). Further, electrophoretic mobility shift assay (EMSA) was performed to confirm the binding of Elk-1 on this motif. Double-stranded radiolabeled oligonucleotides harboring the sequence of an Elk-1-binding motif in the promoter region of 9E3 cCAF gene as a positive control and AIRE promoter as test was used for EMSA and Elk-1 bound to this response element in the AIRE promoter (Fig. 2c, d)^24^. Furthermore, we generated a mutant in which the Elk-1-binding site was mutated in the core motif which abolishes its binding to the AIRE promoter (Fig. 2e). However, Elk-1 expression levels were comparatively similar in both the LNCaP and PC3 cell types (Fig. 2f). To further verify the specificity of this binding and to prove our hypothesis that Elk-1 might be specifically regulating AIRE in PC3 cells without AR, we performed a chromatin immunoprecipitation (ChIP) assay using anti-Elk-1 antibody in both the PC3 and LNCaP cells. PCR amplification with primers showed that Elk-1 interacted with the binding site only in PC3 cells but shows no binding in LNCaP cells as analyzed by fold enrichment (Fig. 2g, h). Similarly, this AIRE dysregulation was also examined in DU145 and VCaP cells as well by ChIP assay (Supplementary Fig. 2d, e). This gives a clue that AR might be interfering with the Elk-1 direct binding on AIRE promoter in LNCaP and VCaP cells. Expression analysis of AIRE was done in the presence of dihydrotestosterone and enzalutamide which acts as an agonist and antagonist for AR, respectively. AIRE expression was increased in LNCaP cells upon agonist treatment and decreased upon antagonist treatment and remained unchanged in PC3 cells. (Fig. 2i–l). We assume that Elk-1 might be responsible for regulating AIRE in PC3 and DU145 cells solely without the AR whereas in LNCaP and VCaP cells it requires making a complex with AR for regulating AIRE. Future studies will take into account the detailed analysis of transcriptional complex regulating the AIRE promoter in varied prostate cancer cell lines to regulate the prostate cancer progression.

### IL-6 is a direct target gene of AIRE

**Fig. 3:**
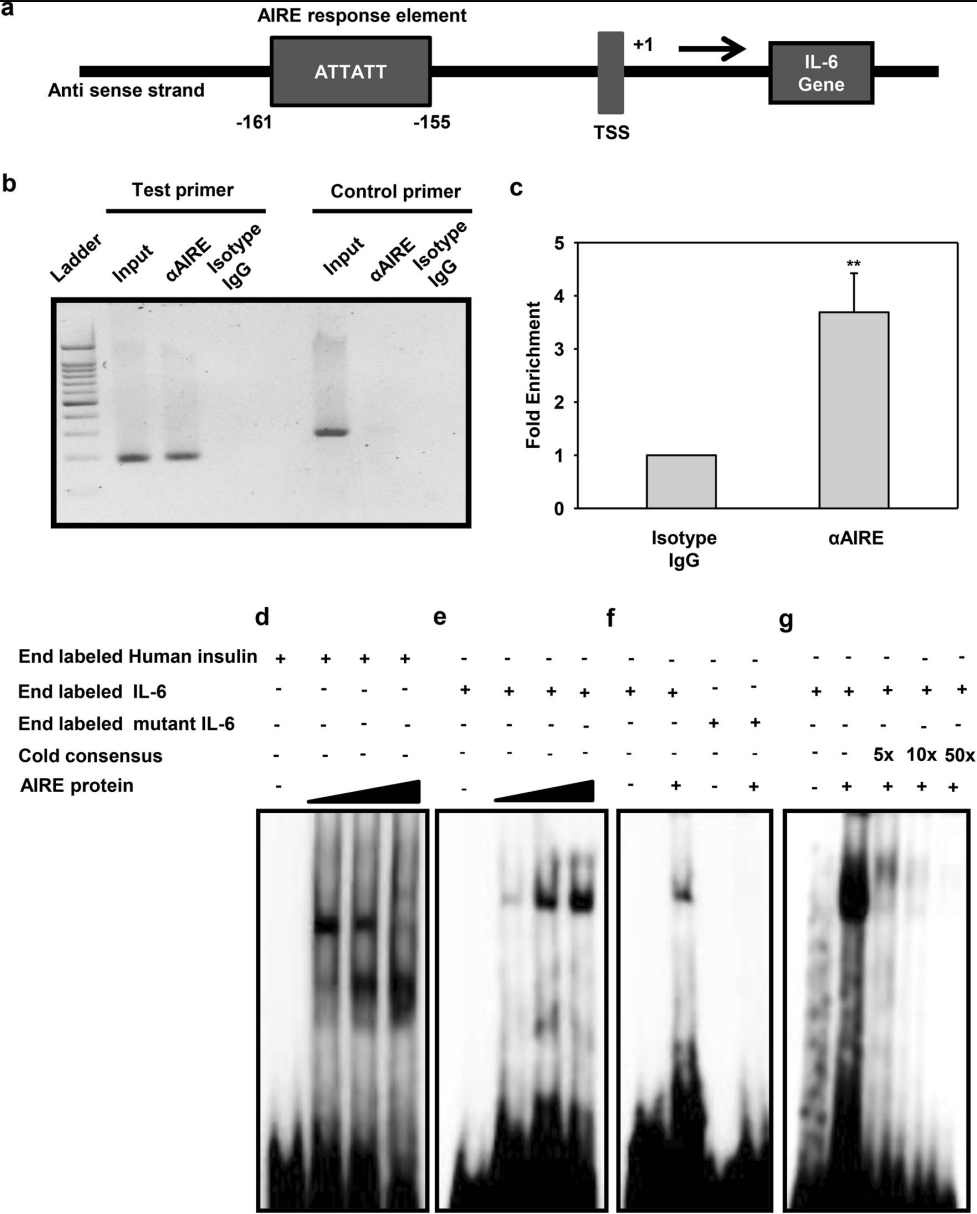
IL-6 is a direct target gene of AIRE. **a** A pictorial illustration of the IL-6 promoter having a conserved motif for AIRE. **b** ChIP analysis of AIRE binding at the human IL-6 promoter region using chromatin isolated from PC3 cells. Crosslinked lysates were immunoprecipitated with ChIP-grade AIRE antibody or isotype control antibody. Eluted DNA proceeded for PCR with specific primer pair covering the region of the IL-6 gene promoter or the GAPDH gene promoter. **c** PCRs with non-immunoprecipitated DNA (input) or without DNA (α-IgG) or) were also performed to analyze the fold enrichment. **d** EMSA analysis using radiolabeled oligonucleotide containing the sequence for AIRE binding on Insulin regulatory region and **e** IL-6 regulatory region in the presence of increasing concentration of in vitro-translated AIRE. **f** EMSA was performed using radiolabeled oligonucleotide containing the sequence for the wild-type IL-6 regulatory region and mutant in which the core motif was mutated. **g** A competition experiment was performed by adding 5-, 10-, and 50-fold excess of cold unlabeled oligonucleotide containing the sequence for consensus IL-6 AIRE response element. Asterisks represent significant differences (**p < 0.005). Data are representative (**b**, **d–g**) and average (**c**) from three independent biological experiments performed in triplicate (mean ± s.d.)

AIRE regulates the expression of its target genes by binding to the conserved motifs on their promoters in dimer and tetramer forms^25^. To determine whether AIRE modulates IL-6 expression at the genomic level, we extracted the promoter sequence of IL-6 gene manually from the NCBI and examined for the AIRE-binding site. We found a putative AIRE-binding site in the proximal region of the IL-6 promoter (Fig. 3a). The binding of AIRE on this site was confirmed by ChIP using anti-AIRE antibody in PC3 cells. PCR amplification with primers showed that AIRE interacted with the binding site. GAPDH served as a negative control. No amplification was observed in DNA isolates with control primers which further validated that the binding and amplification was specific (Fig. 3b, c). To further verify AIRE can bind to the IL-6 promoter additionally, EMSA was performed with a double-stranded radiolabeled oligonucleotide containing the AIRE-binding motif in the region of insulin gene as a positive control and IL-6 promoter (Fig. 3d, e)^26^. Further, we generated mutant to determine the functional relevance of the nucleotides. We observed that binding was totally obliterated when the mutation was done in the core motif (Fig. 3f). Furthermore, the binding was proved specific, as it could compete with unlabeled cold consensus probe (Fig. 3g). To confirm that AIRE is not directly regulating IL-6 promoter in androgen-dependent cells, we also performed ChIP using anti-AIRE antibody in LNCaP cells and we did not observe any binding of AIRE on the IL-6 promoter as analyzed by the fold enrichment (Supplementary Fig. 2c).

### Comparative phylogenetic analysis of the IL-6 promoter with the AIRE-binding motif

**Fig. 4:**
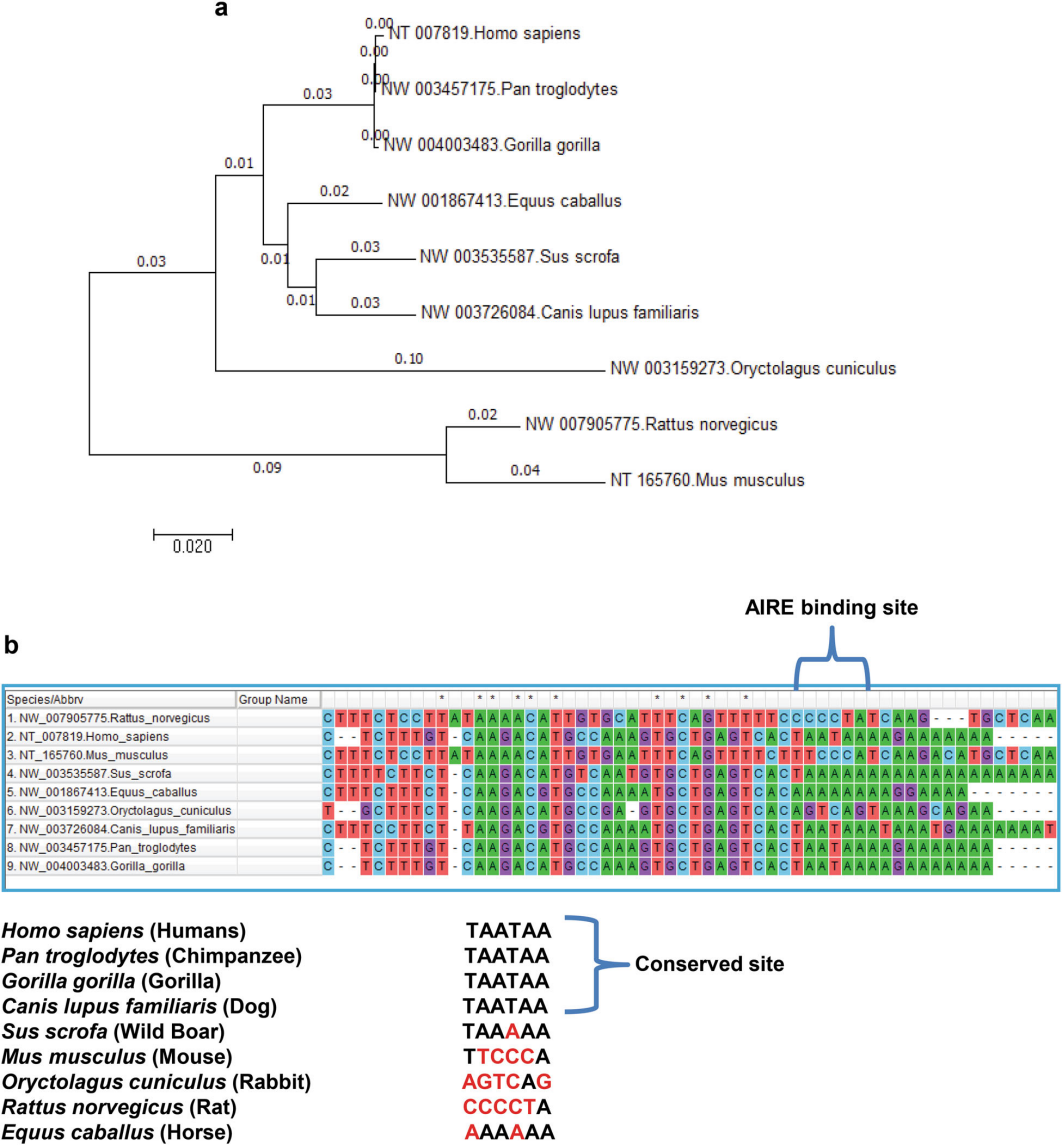
Comparative analysis of IL-6 promoter containing AIRE response element. **a** Phylogenetic tree construction of IL-6 promoters containing the conserved motif for AIRE by MEGA5 (Molecular Evolutionary Genetic Analysis) software using Neighbor-Joining (NJ) method, with 1000 bootstrap replicates. **b** Multiple pairwise sequence alignment of IL-6 promoters encompassing AIRE-binding motif using ClustalW2 among various mammalian species demonstrated heterogeneity in the wild boar IL-6 promoter. Asterisks represent significant differences (*p < 0.05). Core motifs disruptions have been highlighted in red

On the basis of the above observation which confirmed IL-6 as a target gene of AIRE in humans, we were intrigued to analyze the IL-6 gene promoter for the binding site of AIRE in other species as well. And to our consternation conserved motif for AIRE binding was found in other species also. The evolutionary relationship between the IL-6 promoter sequences of nine species comprising putative AIRE motif was also studied by multiple pairwise alignment and phylogenetic tree construction (Fig. 4a, b).

### AIRE rescues PC3 cells from anticancer drug-induced cell death and enhances their invasiveness

**Fig. 5:**
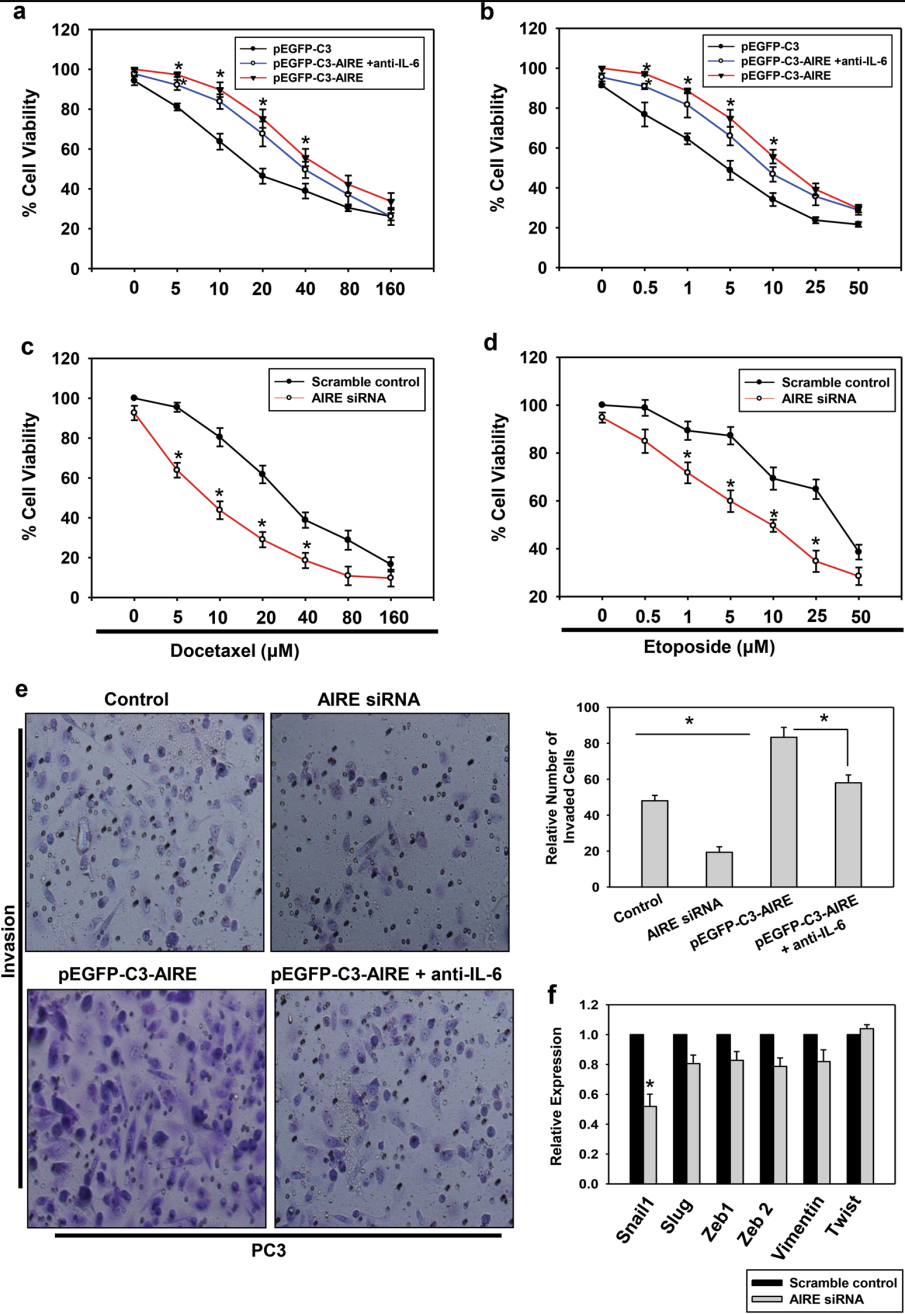
AIRE prevents PC3 cells from drug-induced cell death and increases the invasiveness of cells. **a**, **b** PC3 cells transfected with empty vector, AIRE vector, and AIRE vector with anti-IL-6 antibody or **c**, **d** scrambled RNA and AIRE siRNA were cultured with indicated concentrations of Docetaxel and Etoposide. After 72 h, cell proliferation was measured by MTT assay. IL-6 inhibitory antibody inhibits the AIRE induced cell survival. **e** Invasion assay in matrigel-coated chambers of PC3 cells transfected with control vector, AIRE expression vector, AIRE siRNA, and AIRE expression vector with anti-IL-6 antibody. Representative high-power fields (HPFs) (×200) were photographed and the number of cells that had invaded was counted in five HPFs using the ImageJ® free software. Bar graphs represent the invasion index as the mean ± s.d. of cells counted per-field of three independent experiments. **f** qRT-PCR analysis of various EMT genes in siRNA-mediated knockdown background of AIRE. Asterisks represent significant differences (*p < 0.05). Data are average and representative from three independent biological experiments performed in triplicate (mean ± s.d.)

In order to delineate the effect of AIRE on cell death and viability, we performed MTT assay which is a characteristic of cell metabolic activity. PC3 cells transfected with either pEGFP-C3 and pEGFP-C3-AIRE or scramble control or AIRE siRNA were treated with docetaxel (0–160 μM) and etoposide (0–50 μM) and the cell viability was measured by MTT assay. It was observed that PC3 cells ectopically expressing AIRE were rescued from dose-dependent inhibition of cell proliferation (Fig. 5a, b). On the contrary, PC3 cells with AIRE knockdown background were more sensitive to drug-induced cell death as compared to scramble control transfected cells (Fig. 5c, d). Further, when AIRE-overexpressed cells are pretreated with anti-IL-6 antibody, they exhibited decrease in cell viability as compared to the AIRE-overexpressed cells alone. To further study the possible role of AIRE in the invasion and migration of prostate cancer cells, PC3 cells were transfected with empty vector, AIRE expression vector, or AIRE siRNA. Differences in invasion between control PC3 cells, AIRE-expressing cells, and AIRE knockdown cells were analyzed using a Matrigel invasion assay. The results show that AIRE-expressing PC3 cells were significantly more invasive than the empty vector controls. The addition of neutralizing IL-6 antibody to the cells transfected with AIRE expression vector resulted in decreased invasion of cells. In addition, results revealed that control transfected cells were more invasive in comparison to AIRE knockdown cells (Fig. 5e). In order to confirm the phenomenon of cell invasion, Matrigel invasion assay was also performed in another androgen-independent cell line (DU145) (Supplementary Fig. 4a). Further, to expand these findings to androgen-dependent cells, LNCaP cells were also transfected with the empty vector, AIRE expression vector, and AIRE siRNA and tested for invasion capacity. Similar to the results described above, LNCaP cells expressing AIRE exhibited increased invasion when compared with the control LNCaP cells but show a very low invasion index as compared to PC3 and DU145 cells (Supplementary Fig. 3a). Expression levels of various EMT markers were also checked by qRT-PCR analysis in PC3 cells knockdown with AIRE. We observed a marginal decrease in expression levels of slug, vimentin, ZEB1, and ZEB2 but we observed a significant decrease in the expression of Snail-1. There was no change observed in the expression of twist (Fig. 5f). EMT markers expression levels were also checked in DU145 cells (Supplementary Fig. 4b).

### AIRE induced IL-6 and PGE2 skews monocytes polarization to M2 type

**Fig. 6:**
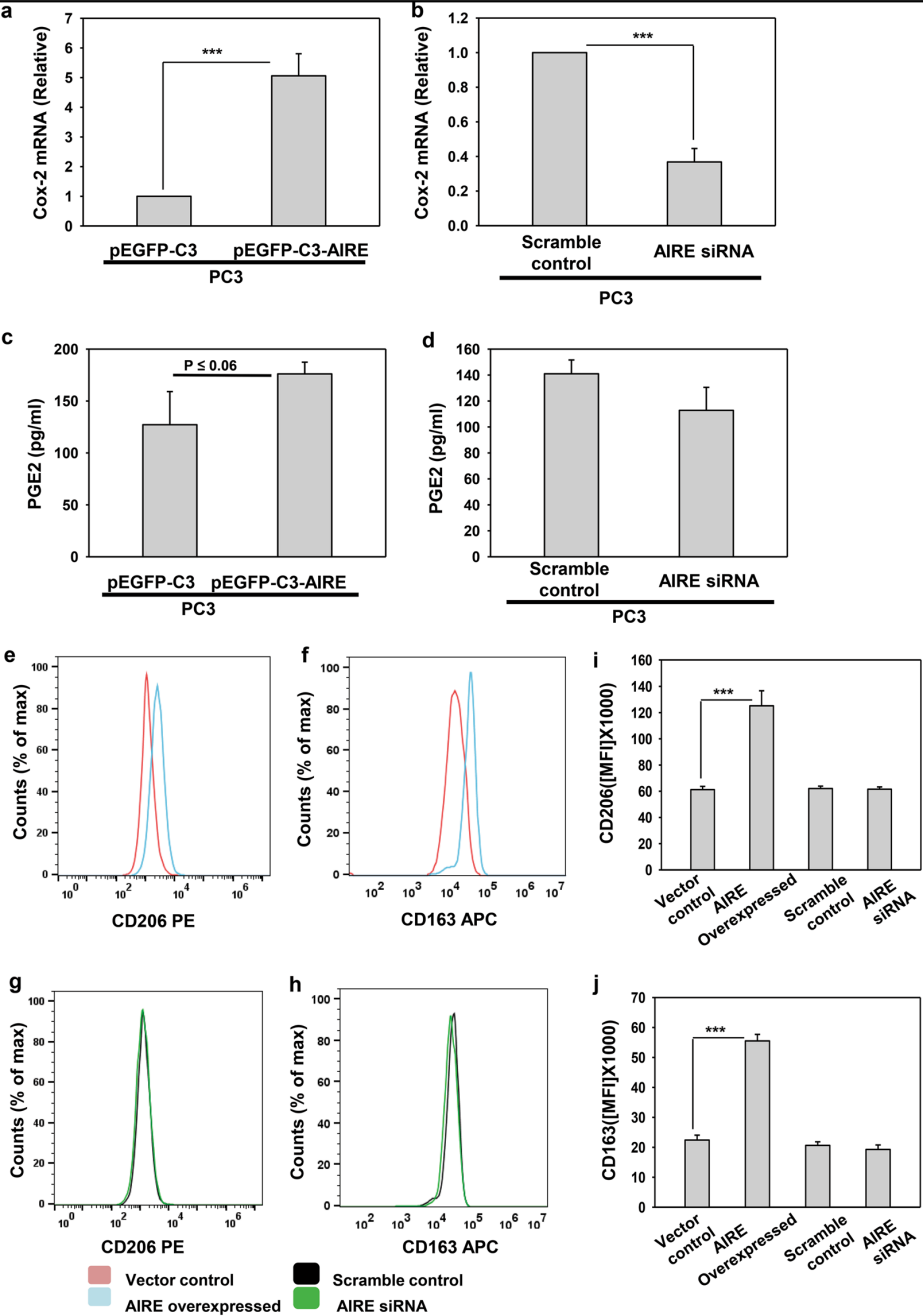
AIRE polarize peripheral blood monocytes towards M2 phenotype by stimulating IL-6 and COX-2/PGE2 expression in PC3 cells. **a**, **b** qRT-PCR analysis of COX-2 in PC3 cells overexpressed with AIRE and knock down with AIRE. **c**, **d** PGE2 ELISA analysis in PC3 cells ectopically expressed with AIRE and in the knockdown background of AIRE. **e**, **f** The polarization status of human monocytes after being treated with conditioned media from PC3 cells overexpressed with AIRE and **g**, **h** knockdown with AIRE was detected by flow cytometric analysis for CD206 and CD163. **i**, **j** Bar graphs represent the mean fluorescence intensity (MFI). Asterisks represent significant differences (***p < 0.001). Data represent the average (**a**–**d**, **i, j**) and representative (**e**–**h**) from three independent biological experiments performed in triplicate (mean ± s.d.)

The phenotype of macrophages derived from monocytes recruited to tumor site depends on the cues in the local tumor milieu. Factors secreted by cancer cells that have been implicated in the polarization of monocytes in the tumor microenvironment include IL-6, IL-10, TGF-β, and prostaglandins (PGE2)^27^. Classical dogma for prostaglandin synthesis follows a chronological order in which arachidonic acid, dihomo-γ-linolenic acid, or eicosapentaenoic acid are oxidized by cyclooxygenases (COX-1 and COX-2) and terminal prostaglandin synthases. In chronic inflammation scenarios, prostaglandin levels are increased by COX-2 upon stimulation^28–30^. So, it was obvious to look if AIRE can modulate COX-2 expression also. We found that AIRE-transfected cells have more COX-2 mRNA levels whereas AIRE knockdown leads to decreased COX-2 mRNA levels (Fig. 6a, b). Further, we analyzed the supernatant from these transfected cells for PGE2 secretion also and we found that AIRE regulating COX-2 expression increases PGE2 secretion as well but when we knockdown AIRE PGE2 secretion is not decreased as much (Fig. 6c, d). This implies that PGE2 is not solely regulated by AIRE through COX-2 but AIRE may assist other transcription factors in its increased production and secretion. It has been shown that dendritic cells differentiation was held back or even skewed towards the tolerogenic M2 macrophages by tumor-derived PGE2 and IL-6^31^. These two inflammatory mediators are also known to polarize the monocytes to M2 macrophages and impart resistance to therapy in gynecological cancers^32^. IL-6 along with CCL2 has been shown to induce the M2 polarization of the peripheral blood-derived monocytes (PBMCs)^33^. As previously shown here, AIRE is significantly regulating IL-6 expression so we investigated the influence of AIRE on monocyte differentiation in tumor microenvironment. We showed that PBMCs incline their differentiation toward M2 type when they were in vitro cultured with the prostate cancer cell supernatant from AIRE-overexpressing cells. We observed no change in the M2 marker expression when we cultured the PBMCs with the supernatant from AIRE knockdown PC3 cells. The M2 phenotype of cells was confirmed by flow cytometry analysis and these cells show increased expression of M2 markers CD206 and CD163 (Fig. 6e–j). Although both the neutralization of IL-6 and inhibition of COX-2 have been reported to prevent the M2 differentiation from monocytes, IL-6 which gets transcriptionally regulated by AIRE might contribute significantly to M2 phenotype of the monocytes in comparison to COX-2.

### AIRE modulates prostate tumorigenesis in mouse model of prostate cancer

**Fig. 7:**
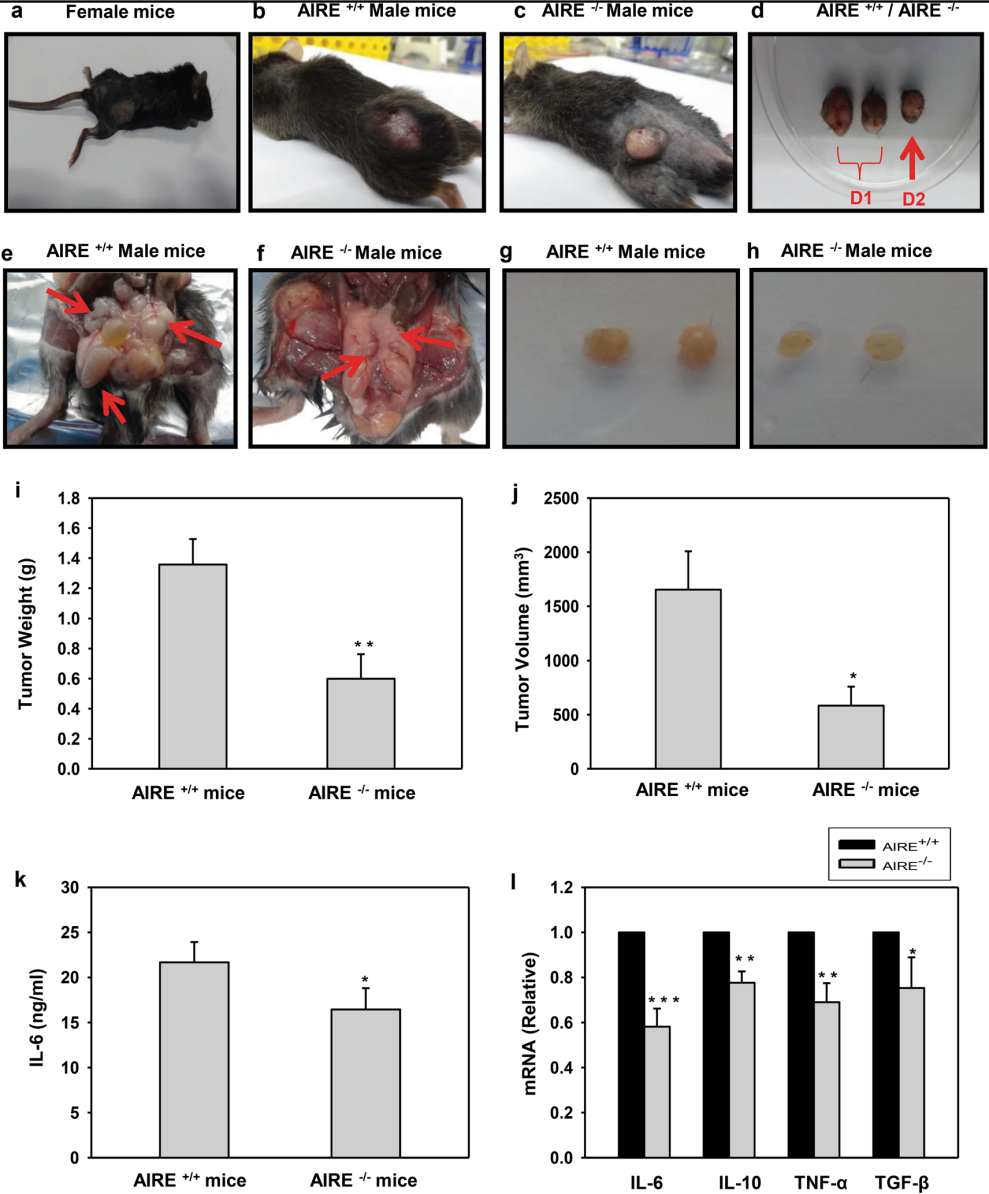
In vivo tumor generation. **a** A mouse from female group presents no visible macroscopic tumor in the flank as a result of the subcutaneous injection of TRAMP-C1 cells and acts as a negative control **b** AIRE^+/+^ male mouse forms a well-delimiting big palpable tumor and is visible on the left flank (arrow) (40 days p.i.). **c** A small visible tumor (arrow) is present in the left flank of the mouse from AIRE^−/−^ male group (55 days p.i.). **d** For clarity, tumor in the extracted anatomic piece, have been highlighted in the box, i.e. D1 from the AIRE^+/+^ male mice group and D2 from the AIRE^−/−^ male mice group. **e** Upon dissecting AIRE^+/+^ male mouse (40 days p.i.) local inflammation is seen in seminal vesicles, abdominal cavity with more prominent growth, and inflammation of adjacent tissues (**f**) whereas upon dissecting AIRE^−/−^ male mouse (55 days p.i.) constricted anatomy is observed with no inflamed organs. **g** Lymphadenopathy is observed in pelvic lymph nodes isolated from AIRE^+/+^ male mouse **h** whereas lymph nodes appear normal in size in AIRE^−/−^ male mice. **i** Tumor weight and **j** tumor volume of the extracted tumors were assessed. **k** IL-6 levels in the mice sera were measured by ELISA. **l** Expression analysis of cytokines in intratumoral lysates by qRT-PCR. Asterisks represent significant differences (***p < 0.0005, **p < 0.005, *p < 0.05). Data represent the representative (**a–h**) and average (**i–l**) from three independent biological experiments (*n* = 4–5 animals per group) performed in triplicate (mean ± s.d.)

To ascertain the pathological significance of AIRE in contributing to metastasis, we evaluated in vivo tumor generation and metastasis in the mouse model. Here, we required an allograft mouse model which is generated by using the tumor cell lines obtained from the identical genetic background as we needed to generate tumor model in AIRE^+/+^ and AIRE^−/−^ mice. TRAMP-C1 is a hormone-independent PCa cell line derived from a TRAMP tumor and is widely used to establish orthotopic and subcutaneous murine model of prostate cancer. The TRAMP model has been proven helpful in assessing novel therapeutic approaches for PCa^34^. 5 × 10^6^ tumor cells in 0.1 ml of media were injected subcutaneously into the flanks of 8-week-old immunocompetent wild-type (WT) female mice, male AIRE^+/+^ and male AIRE^−/−^ mice and tumor generation was monitored weekly. Subcutaneous injection of TRAMP-C1 cells resulted in no visible tumor in female mice (Fig. 7a), big palpable benign tumors in male AIRE^+/+^ mice after 40 days of initial injection whereas to our surprise we observed small localized tumor growth in case of male AIRE^−/−^ mice after 55 days postinjection (Fig. 7b, c). Tumors have been excised in the anatomic piece D1 from male AIRE^+/+^ mice and D2 from male AIRE^−/−^ mice (Fig. 7d). Upon dissecting the mice, local inflammation in the prostate, seminal vesicles, and the abdominal cavity was observed in AIRE^+/+^ mice whereas, in contrast, AIRE^−/−^ mice had an organized constricted anatomy with no such inflammation (Fig. 7e, f). Lymphadenopathy is generally associated with prostate cancer metastasis and as predictable lymph nodes isolated were bigger in size due to inflammation in AIRE^+/+^ mice as compared to lymph nodes from AIRE^−/−^ mice (Fig. 7g, h)^35,36^. Tumor weight and volume have been measured (Fig. 7i, j). Further, IL-6 levels in the mice sera have also been measured (Fig. 7k). Cytokines expression has also been checked in the intratumoral lysates (Fig. 7l). Comparative analysis in Fig. 3 shows discrepancy in the shown binding site of AIRE on mIL-6 promoter. So, we analyzed the mIL-6 promoter for any other AIRE-binding site mediating all these effects due to IL-6 upregulation. We found an AIRE-binding site in the proximal promoter of the mIL-6 (Supplementary Fig. 5a). We performed ChIP assay with anti-AIRE antibody in TRAMP-C1 cells and we found that AIRE regulates mIL-6 promoter through this binding site (Supplementary Fig. 5b). Further, we also performed EMSA with the wild type and mutant radiolabeled probes (Supplementary Fig. 5c, d). This enticing observation clearly explained that AIRE might further lead to metastasis in AIRE^+/+^ mice by inducing malignancy factor IL-6.

## Discussion

Decades of research have converged on genetic abnormalities which instigate the prostate cancer but overwhelming evidence has emphasized the need of tumor microenvironment in deciding cancer cells behavior to drive and promote metastasis^6^. Targeting the intercellular communication in the tumor microenvironment which is driven by various factors like cytokines, chemokines, matrix remodeling enzymes, and other growth factors may prove to be a better therapeutic approach^37,38^. However, the release of these secretory factors is accompanied by peculiar gene expression program and is not completely understood. Here, in this study, we have found AIRE as a host transcription factor which promotes tumor by acting as a transcriptional inducer of the IL-6 gene (Fig. 3 and Supplementary Fig. 5). AIRE-binding motif on IL-6 promoter has been found well conserved in higher primates through phylogenetic analysis confirming its functional relevance throughout evolution (Fig. 4). Moreover, AIRE overexpression in PC3 prostate cancer cells resulted in inhibition of drug-induced cell death thereby promoting cell survival and it also enhances the invasiveness of cancer cells which is a pre-requisite phenomenon leading to metastasis (Fig. 5). Furthermore, PBMCs cultured with PC3 conditioned media polarized the cells to M2 phenotype (Fig. 6). Overall, this strongly supports a pro-cancerous role of AIRE in prostate cancer.

The human immune system shows a major sexual dimorphism in infectious diseases and autoimmune disorders^39–42^. Although there occurs a clear role of hormonal and environmental factors contributing to this but molecular interactions have not been addressed so far. Given the role of AIRE in the modulation of autoimmune diseases in a sex hormone-dependent manner, our goal was to unravel its role in other diseases. Studies have shown that AIRE dampens the antitumor immune response in melanoma and sarcomas leading to cancer progression^12,43^. Another group demonstrated that AIRE-expressing medullary thymic epithelial cells (mTECs) undergo maturation process by receptor activator of NF-κB (RANK)–RANK ligand signaling pathway and RANKL blockade leads to enhanced tumor protection against B16 melanoma^44,45^. Expression of intermediate filament protein keratin K17 is found to be upregulated in skin tumors and is responsible for induction of AIRE in tumor keratinocytes which is required for the onset of Gli-2 mediated skin tumorigenesis^46^.

Cancers can be hormone sensitive and there is a striking variation in the risk of hormone-dependent breast and thyroid cancers between males and females, more likely due to their endogenous hormonal profile^47^. Understanding how this interaction may be monitored and modulated could propose new hormone-based interventions to reduce cancer risk. According to the Global Cancer Statistics, most common death causing cancers in men and women are prostate cancer and breast cancer respectively and both are largely influenced by sexual hormones. Recent evidence has shown that AIRE is being regulated genomically by androgens and epigenetically by estrogens^10,11^. AIRE overexpression in estrogen-sensitive breast cancer cells leads to cell cycle arrest and apoptosis defining its anti-cancerous role^13^. AIRE regulates T regulatory cells (Tregs) isolated originally from prostate cancer, which may get co-opted by tumors developing in the organ posing its immunoregulatory role in prostate cancer^48^. Excavating the intricate role of AIRE in androgen-dependent and -independent prostate cancer opened new avenues in the field of molecular regulation of AIRE and the causation. AIRE expression was found more in PC3 cells as compared to LNCaP cells (Fig.1a, b) and it is being regulated constitutively by Elk-1 in PC3 cells which are devoid of the AR (Fig. 2h). AIRE being a regulator of IL-6 malignancy gene shows lymphadenopathy and inflammation in AIRE^+/+^ mice and only a small benign tumor is observed AIRE^−/−^ mice (Fig. 7).

**Fig. 8:**
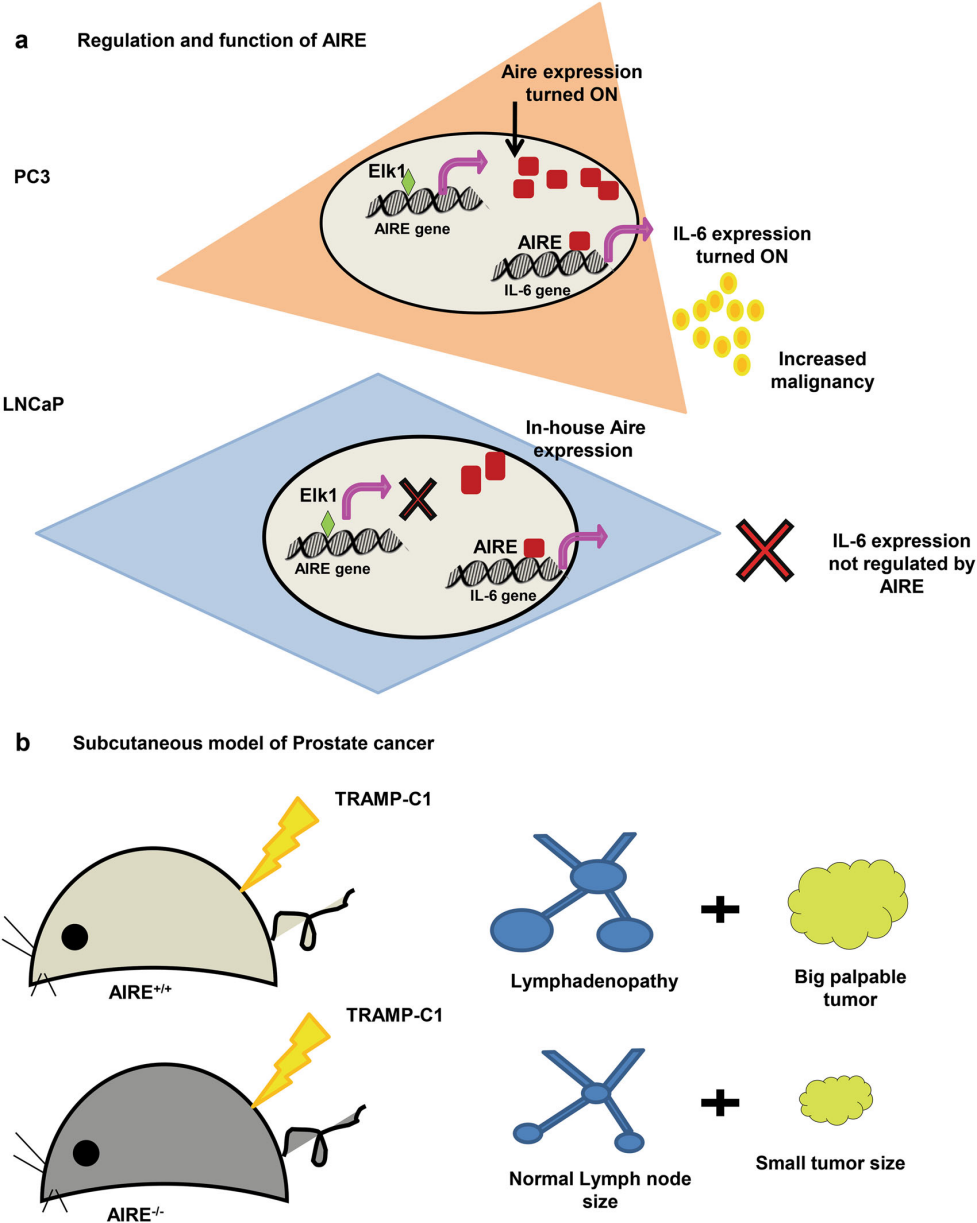
Schematic representation featuring the role of AIRE in prostate cancer progression. **a** AIRE transcriptionally regulates IL-6 gene in PC3 cells and not in LNCaP cells. Elk-1 is able to promote AIRE expression by directly binding to its gene promoter specifically in PC3 cells and not in LNCaP cells. **b** Subcutaneous TRAMP-C1 mouse model of prostate cancer in male AIRE^+/+^ mice forms a big palpable tumor 40 days (p.i.) and presents lymphadenopathy whereas male AIRE^−/−^ mice forms a small benign tumor 55 days (p.i.) and lymph nodes are normal in size. Female mice act as a negative control with no tumor development

The disparity in AIRE expression among androgen-sensitive and -insensitive PCa cells defines its specific role in metastatic PCa and this expression analysis in patients may help in understanding the degree of PCa risk. Largely, the level of AIRE expression and its regulation affect the outcome of prostate cancer by modulating the confined tumor milieu (Fig. 8). Overall, this field-advancing study revealed AIRE-associated molecular regulation of prostate cancer immunity. Therefore, along with hormone ablation therapy inclusion of an AIRE antagonist is likely to be a promising strategy for prostate cancer patients.

## Materials and methods

### Animals and ethics statement

Aire (B6.129S2-*Aire*^*tm1.1Doi*^/J)^–/–^ mice were procured from the Jackson Laboratory. All animals were housed in the pathogen-free animal facility at Institute of Microbial Technology (IMTECH) according to the guidelines of the Institutional Animal Ethics Committee (No. 55/1999/CPCSEA), Ministry of Environment and Forests, Government of India. F1 heterozygous males and females were mated to produce F2 wild type, heterozygous, and homozygous null mutants. Mice were genotyped according to the instructions on the Jackson website. A cohort of age- and sex-matched 7–8-week-old male Aire^–/–^ and Aire^+/+^ and female Aire^+/+^ littermates used for the experiments were procured from the institute’s animal facility.

### Cell culture and differentiation

PC3, LNCaP, DU145, VCaP (human), and TRAMP-C1 (mouse) prostate cancer cell lines were purchased from ATCC (Rockville, MD, USA). PC3, LNCaP, DU145, and VCaP cells were maintained in F12K nutrient, RPMI-1640, MEM, and DMEM media, respectively, supplemented with 10% fetal bovine serum (FBS) and 1% penicillin–streptomycin (Pen-Strep; Gibco). TRAMP-C1 cells were maintained in DMEM media supplemented with 0.005 mg/ml bovine insulin and 10 nM dehydroisoandrosterone (90%); FBS, 5%; Nu-Serum IV, 5%. Peripheral blood was taken from healthy individuals. PBMCs were isolated by the Ficoll paque density gradient centrifugation method and were differentiated as described earlier^49^. The project was approved by the Ethics Committee of the Government Medical College and Hospital, Sector 32, and the Ethics and Biosafety Committee of IMTECH, Sector 39A (Chandigarh, India).

### Chromatin immunoprecipitation

ChIP was performed according to the protocol mentioned earlier^50^. The following primers were used in the ChIP assay: Human GAPDH promoter forward 5′-CGGTGCGTGCCCAGTTG-3′ and reverse 5′-GCGACGCAAAAGAAGATG-3′, hIL-6 promoter AIRE response element forward 5′-GTCAAGACATGCCAAAGTGC-3′ and reverse 5′-GGTGGGGCTGATTGGAAA-3′, mIL-6 promoter AIRE response element forward 5′-GCCTCAAGGATGACTTAAGC-3′ and reverse 5′-GAATTGACTATCGTTCTTGGTG-3′. Primers corresponding to Elk-1 binding on AIRE promoter are F 5′-ACCACAGCCTCAGCATCATT-3′ R 5′-TTCCTAGGACCAGTGACAGA-3′.

### Electrophoretic mobility shift assay

Oligonucleotides were annealed and end-labeled using γ^32^-P-ATP and T4-polynucleotide kinase. AIRE protein was prepared in vitro by using the 1-Step Human Coupled IVT Kit (Thermo Scientific) according to the manufacturer’s instructions. The wild-type oligonucleotide corresponding to AIRE-binding site −155 to −161 on hIL-6 promoter has a sequence 5′-TTTTCTTTTATTAGTGACTCAGCA-3′ and the mutant probe in which the binding motif has been mutated has a sequence 5′-TTTTCTCATCGTGTGACTCAG-3′. The wild-type oligonucleotide corresponding to AIRE-binding site −76 to −81 on mIL-6 promoter has a sequence 5′-CTCATATTTATTAGGAGTCAACT-3′ and the mutant probe has a sequence 5′-CTCATATGGCGGCGGAGTCAACT-3′. The wild-type probe corresponding to the Elk-1-binding site −778 to −785 on AIRE promoter has a sequence 5′-AGATGCCCCCGGAAGCTCCTGTCCAG-3′ and the mutant probe has a sequence 5′-AGATGCCCCCTTCCTCTCCTGTC-3′. Protein–DNA interaction was performed as described earlier^50^.

### Measurement of cytokine levels by ELISA

Cytokines in cell-culture supernatants and mouse serum were quantified by commercial ELISA kits specific for human and mouse IL-6 (BD Biosciences) and PGE2 (Prostaglandin E2 Parameter Assay Kit (R&D Systems)) in accordance with the manufacturer’s instructions.

### In vivo tumor generation

Subcutaneous tumors were generated by injecting TRAMP-C1 cells (5 × 10^6^ cells/0.2 ml media) subcutaneously into the flank of 8-week-old mice. Animals were monitored twice weekly for tumor development. Mice were sacrificed 40–60 days postinjection (p.i.).

### Statistical analysis

Sigmaplot was used for performing the statistical analysis. Results are expressed as the mean and standard deviation unless otherwise mentioned. Unpaired Student’s *t*-tests were done to obtain *p-*values. Statistical significance was established at *p *<* 0.05, **p *<* 0.01, ***p *<* 0.001

## Electronic supplementary material

Supplementary Information

Supplementary Figure 1

Supplementary Figure 2

Supplementary Figure 3

Supplementary Figure 4

Supplementary Figure 5

## References

[1] Siegel RL, Miller KD, Jemal A (2015). Cancer statistics, 2015. CA Cancer J. Clin..

[2] Hellerstedt BA, Pienta KJ (2002). The current state of hormonal therapy for prostate cancer. CA A Cancer J. Clin..

[3] Feldman BJ, Feldman D (2001). The development of androgen-independent prostate cancer. Nat. Rev. Cancer.

[4] Kahn B, Collazo J, Kyprianou N (2014). Androgen receptor as a driver of therapeutic resistance in advanced prostate cancer. Int. J. Biol. Sci..

[5] Saraon P, Drabovich AP, Jarvi KA, Diamandis EP (2014). Mechanisms of androgen-independent prostate cancer. EJIFCC.

[6] Shiao SL, Chu GC, Chung LW (2016). Regulation of prostate cancer progression by the tumor microenvironment. Cancer Lett..

[7] Barron DA, Rowley DR (2012). The reactive stroma microenvironment and prostate cancer progression. Endocr. Relat. Cancer.

[8] Zhou Y, Bolton EC, Jones JO (2015). Androgens and androgen receptor signaling in prostate tumorigenesis. J. Mol. Endocrinol..

[9] Bhagwat AS, Vakoc CR (2015). Targeting transcription factors in cancer. Trends Cancer.

[10] Zhu ML (2016). Sex bias in CNS autoimmune disease mediated by androgen control of autoimmune regulator. Nat. Commun..

[11] Dragin N (2016). Estrogen-mediated downregulation of AIRE influences sexual dimorphism in autoimmune diseases. J. Clin. Invest..

[12] Zhu ML, Nagavalli A, Su MA (2013). Aire deficiency promotes TRP-1-specific immune rejection of melanoma. Cancer Res..

[13] Bianchi F (2016). Expression and prognostic significance of the autoimmune regulator gene in breast cancer cells. Cell Cycle.

[14] Eldershaw SA, Sansom DM, Narendran P (2011). Expression and function of the autoimmune regulator (Aire) gene in non-thymic tissue. Clin. Exp. Immunol..

[15] Russell PJ, Kingsley EA (2003). Human prostate cancer cell lines. Methods Mol. Med..

[16] Dranoff G (2004). Cytokines in cancer pathogenesis and cancer therapy. Nat. Rev. Cancer.

[17] Landskron G, De la Fuente M, Thuwajit P, Thuwajit C, Hermoso MA (2014). Chronic inflammation and cytokines in the tumor microenvironment. J. Immunol. Res..

[18] Culig Z (2014). Proinflammatory cytokine interleukin-6 in prostate carcinogenesis. Am. J. Clin. Exp. Urol..

[19] Nguyen DP, Li J, Tewari AK (2014). Inflammation and prostate cancer: the role of interleukin 6 (IL-6). BJU Int..

[20] Murumagi A, Silvennoinen O, Peterson P (2006). Ets transcription factors regulate AIRE gene promoter. Biochem. Biophys. Res. Commun..

[21] Rosati R (2016). The amino-terminal domain of the androgen receptor co-opts extracellular signal-regulated kinase (ERK) docking sites in ELK1 protein to induce sustained gene activation that supports prostate cancer cell growth. J. Biol. Chem..

[22] Xiao D, Qu X, Weber HC (2002). GRP receptor-mediated immediate early gene expression and transcription factor Elk-1 activation in prostate cancer cells. Regul. Pept..

[23] Patki M (2013). The ETS domain transcription factor ELK1 directs a critical component of growth signaling by the androgen receptor in prostate cancer cells. J. Biol. Chem..

[24] Li Q, Vaingankar SM, Green HM, Martins-Green M (1999). Activation of the 9E3/cCAF chemokine by phorbol esters occurs via multiple signal transduction pathways that converge to MEK1/ERK2 and activate the Elk1 transcription factor. J. Biol. Chem..

[25] Kumar PG (2001). The autoimmune regulator (AIRE) is a DNA-binding protein. J. Biol. Chem..

[26] Ruan QG (2007). The autoimmune regulator directly controls the expression of genes critical for thymic epithelial function. J. Immunol..

[27] Rhee I (2016). Diverse macrophages polarization in tumor microenvironment. Arch. Pharm. Res..

[28] Chizzolini C, Brembilla NC (2009). Prostaglandin E2: igniting the fire. Immunol. Cell Biol..

[29] Harris SG, Padilla J, Koumas L, Ray D, Phipps RP (2002). Prostaglandins as modulators of immunity. Trends Immunol..

[30] Park JY, Pillinger MH, Abramson SB (2006). Prostaglandin E2 synthesis and secretion: the role of PGE2 synthases. Clin. Immunol..

[31] Heusinkveld M (2011). M2 macrophages induced by prostaglandin E2 and IL-6 from cervical carcinoma are switched to activated M1 macrophages by CD4+ Th1 cells. J. Immunol..

[32] Dijkgraaf EM (2013). Chemotherapy alters monocyte differentiation to favor generation of cancer-supporting M2 macrophages in the tumor microenvironment. Cancer Res..

[33] Roca H (2009). CCL2 and interleukin-6 promote survival of human CD11b+ peripheral blood mononuclear cells and induce M2-type macrophage polarization. J. Biol. Chem..

[34] Hurwitz A. A., Foster B. A., Allison J. P., Greenberg N. M. & Kwon E. D. The TRAMP mouse as a model for prostate cancer. Curr. Protoc. Immunol. (2001); Chapter 20: Unit 20 25. 10.1002/0471142735.im2005s45 18432778

[35] Komeya M, Sahoda T, Sugiura S, Sawada T, Kitami K (2012). A case of metastatic prostate adenocarcinoma to an inguinal lymph node. Cent. Eur. J. Urol..

[36] Kosugi S (2007). Prostate cancer with supraclavicular lymphadenopathy and bulky abdominal tumor. Intern. Med..

[37] Burkholder B (2014). Tumor-induced perturbations of cytokines and immune cell networks. Biochim. Biophys. Acta.

[38] Karlou M, Tzelepi V, Efstathiou E (2010). Therapeutic targeting of the prostate cancer microenvironment. Nat. Rev. Urol..

[39] Ngo ST, Steyn FJ, McCombe PA (2014). Gender differences in autoimmune disease. Front. Neuroendocrinol..

[40] Guerra-Silveira F, Abad-Franch F (2013). Sex bias in infectious disease epidemiology: patterns and processes. PLoS ONE.

[41] Klein SL, Flanagan KL (2016). Sex differences in immune responses. Nat. Rev. Immunol..

[42] Rubtsova K, Marrack P, Rubtsov AV (2015). Sexual dimorphism in autoimmunity. J. Clin. Invest..

[43] Akiyama N (2014). Limitation of immune tolerance-inducing thymic epithelial cell development by Spi-B-mediated negative feedback regulation. J. Exp. Med..

[44] Anderson MS, Su MA (2016). AIRE expands: new roles in immune tolerance and beyond. Nat. Rev. Immunol..

[45] Khan IS (2014). Enhancement of an anti-tumor immune response by transient blockade of central T cell tolerance. J. Exp. Med..

[46] Hobbs RP (2015). Keratin-dependent regulation of Aire and gene expression in skin tumor keratinocytes. Nat. Genet..

[47] Folkerd EJ, Dowsett M (2010). Influence of sex hormones on cancer progression. J. Clin. Oncol..

[48] Malchow S (2013). Aire-dependent thymic development of tumor-associated regulatory T cells. Science.

[49] Chandra V (2013). Human IL10 gene repression by Rev-erbalpha ameliorates Mycobacterium tuberculosis clearance. J. Biol. Chem..

[50] Mahajan S (2015). Nuclear receptor Nr4a2 promotes alternative polarization of macrophages and confers protection in sepsis. J. Biol. Chem..

