# Supplementary material for: AIRE promotes androgen-independent prostate cancer by directly regulating IL-6 and modulating tumor microenvironment
Source: Oncogenesis. 2018 May 25;7(5):43. doi: 10.1038/s41389-018-0053-7 (PMC5968032; doi:10.1038/s41389-018-0053-7)
Supplement: Supplementary file 1 — Supplementary Information [file 41389_2018_53_MOESM1_ESM.docx]

**Supplementary Information**

**Supplementary Methods**

**Transfection and small interfering RNA-mediated silencing**

Plasmids and/or siRNA were transiently transfected into the cells by using Turbofect Transfection Reagent in accordance with manufacturer’s instructions (Thermo Scientific). For overexpression, cells were transfected with pEGFP-C3 and pEGFP-C3-AIRE (4 μg per well in 6-well plates; 0.2 μg per well in 96 well plates) pEGFP-C3-AIRE was kindly provided by Prof. Mitsuru Matsumoto (Tokushima University). For transient silencing, cells were transfected with 60 nM scramble control or a pool of two AIRE-specific small interfering RNA (30 nM each) targeting two uncommon regions of an mRNA (Qiagen) in Opti-MEM and incubated in the CO2 incubator at 37˚C. Overexpression and knockdown of AIRE were observed after 48 hours.

**MTT Assay**

Cell viability was determined by MTT (Sigma-Aldrich, M5655) assay. Cells were cultured in a 96-well plate. After 48 hours of transfection, cells were treated with different concentrations of anti-cancer drugs etoposide and docetaxel (Sigma- Aldrich, 01885 and E1383) and kept for another 72 hours. A 20-μl aliquot of MTT (5 mg/ml in PBS) was added to each well and incubated for 4 hours at 37^0^C. After 4 hours, 50 μl of 20% SDS/50% DMF was added to each well, kept for another 12 hours and the absorbance was read at 570 nm.

**Invasive Assay**

### Transwell inserts of 6.5 mm-diameter (Costar, Cambridge, MA) with the 8 μm-pore membranes coated with Matrigel (Becton Dickinson (BD), Bedford, MA) were used to assess the invasive capacity of prostate cancer cells. Cells were detached, washed and resuspended in serum-free media. A total of 5×10^4^ cells in 0.2 ml were placed in each chamber. 24 hours later the cells in the upper chamber that did not migrate were scraped away gently and adhered cells on the lower surface of the insert were stained with Giemsa stain and photographed.

### Flow cytometry

For flow cytometric analysis antibodies were titrated as required and surface marker expression analysis was performed by surface staining of the cells at a density of 10^5^ for 30 minutes at 4^0^C with PE conjugated anti-CD-206, and APC-conjugated anti-CD163 (eBiosciences/BD Biosciences). Samples were acquired on BD FACS Accuri C6 and analyzed by using FlowJo software.

**RNA isolation and quantitative Real-Time PCR**

Total RNA was isolated from cells by the Trizol method. 1 μg of total RNA was used for complementary DNA (cDNA) synthesis with the Verso cDNA kit (Thermo Scientific) according to manufacturer’s protocol. cDNA was subsequently amplified by qRT-PCR (DyNAmo ColorFlash SYBR Green qPCR Kit, Thermo Scientific) with gene-specific primers. β-actin mRNA was used as an endogenous control. Relative fold change was obtained by using the formula 2^-ΔΔCt^.

**Western blotting**

For immunoblotting, 30 μg of whole cell extract was resolved by SDS-PAGE and transferred to polyvinylidene difluoride (PVDF) membranes (Immobilon-P; Millipore). After that membranes were blocked with 5% BSA or 5% skim milk in Tris-buffered saline (pH 7.4) for 2 hours and incubated overnight with primary antibodies followed by 2 hours incubation with HRP-conjugated secondary antibodies. The blots were visualized with Luminata Forte Western HRP substrate (Millipore).

**Supplementary Figure Legends**

**Supplementary figure 1. Ectopic expression of AIRE and knockdown of AIRE in PC3 cells and expression analysis of AIRE in DU145 and VCaP cells. (a)** qRT-PCR analysis was done for AIRE in PC3 cells transfected with pEGFP-C3 and pEGFP-C3-AIRE and whole cell lysate was prepared from a similar set of cells followed by immunoblotting with anti-GFP. **(b)** qRT-PCR analysis was done for AIRE in PC3 cells transfected with scrambled RNA and AIRE siRNA and whole cell lysate was prepared from a similar set of cells followed by immunoblotting with anti-AIRE. **(c)** qRT-PCR analysis was done for AIRE in VCaP and DU145 cells. **(d)** Whole cell lysate was prepared from VCaP and DU145 cells followed by immunoblotting with anti-AIRE. Asterisks represent significant differences (*** indicates p<0.0005, **p<0.005). Data is representative and average from three independent biological experiments performed in triplicate (mean ± s.d).

**Supplementary figure 2. IL-6 is not regulated by AIRE in LNCaP cells and regulation of AIRE by Elk-1 in LNCaP, DU145 and VCaP cells. (a)** qRT-PCR analysis of IL-6 in LNCaP cells transfected with pEGFP-C3 and pEGFP-C3-AIRE. **(b)** Expression of AIRE in LNCaP cells ectopically expressed with Elk-1. **(c)** ChIP analysis of AIRE binding at the human IL-6 promoter region using chromatin isolated from LNCaP cells. **(d)** ChIP analysis of Elk-1 binding at the human AIRE promoter region using chromatin isolated from DU145 cells. **(e)** ChIP analysis of Elk-1 binding at the human AIRE promoter region using chromatin isolated from VCaP cells. Asterisks represent significant differences (* indicates p<0.05). Data represents average from three independent biological experiments performed in triplicate (mean ± s.d).

**Supplementary figure 3. Invasive assay in Matrigel-coated chambers of LNCaP cells (a)** Invasion assay in Matrigel-coated chambers of LNCaP cells. Representative high power (x200) fields were photographed and the invaded number of the cells were counted in five HPFs using the ImageJ® free software. Bar graphs represent the invasion index as the mean ± s.d of cells counted per-field of three independent biological experiments performed in triplicate

**Supplementary figure 4. Invasive assay in Matrigel-coated chambers of DU145 cells and expression analysis of EMT markers in DU145 cells. (a)** Invasion assay in Matrigel-coated chambers of LNCaP cells. Representative high power (x200) fields were photographed and the invaded number of the cells were counted in five HPFs using the ImageJ® free software. Bar graphs represent the invasion index as the mean ± s.d of cells counted per-field of three independent biological experiments performed in triplicate. **(b)** qRT-PCR analysis of EMT gene markers in DU145 cells transfected with scramble control and AIRE siRNA. Asterisks represent significant differences (*** indicates p<0.0005, *p<0.05). Data in **(b)** represents average from three independent biological experiments performed in triplicate (mean ± s.d).

**Supplementary figure 5. mIL-6 is a direct target gene of mAIRE.** **(a)** A pictorial illustration of the mIL-6 promoter having a conserved motif for AIRE. **(b)** ChIP analysis of AIRE binding at the mIL-6 promoter region using chromatin isolated from TRAMP-C1 cells. Crosslinked lysates were immunoprecipitated with ChIP-grade AIRE antibody or isotype control antibody. Eluted DNA proceeded for PCR with specific primer pair covering the region of the mIL-6 gene promoter. PCRs with non-immunoprecipitated DNA (input) or without DNA (α-IgG) or) were also performed to analyse the fold enrichment. **(c)** EMSA analysis using radiolabeled oligonucleotide containing the sequence for AIRE binding on mIL-6 regulatory region in the presence of increasing concentration of *in-vitro* translated AIRE. **(d)** EMSA was performed using radiolabeled oligonucleotide containing the sequence for the wildtype mIL-6 regulatory region and mutant in which the core motif was mutated. Asterisks represent significant differences (*** indicates p<0.0005). Data is representative **(c,d)** and average **(b)** from three independent biological experiments performed in triplicate (mean ± s.d).
