# Supplementary figures and images for: AIRE promotes androgen-independent prostate cancer by directly regulating IL-6 and modulating tumor microenvironment
Source: Oncogenesis. 2018 May 25;7(5):43. doi: 10.1038/s41389-018-0053-7 (PMC5968032; doi:10.1038/s41389-018-0053-7)

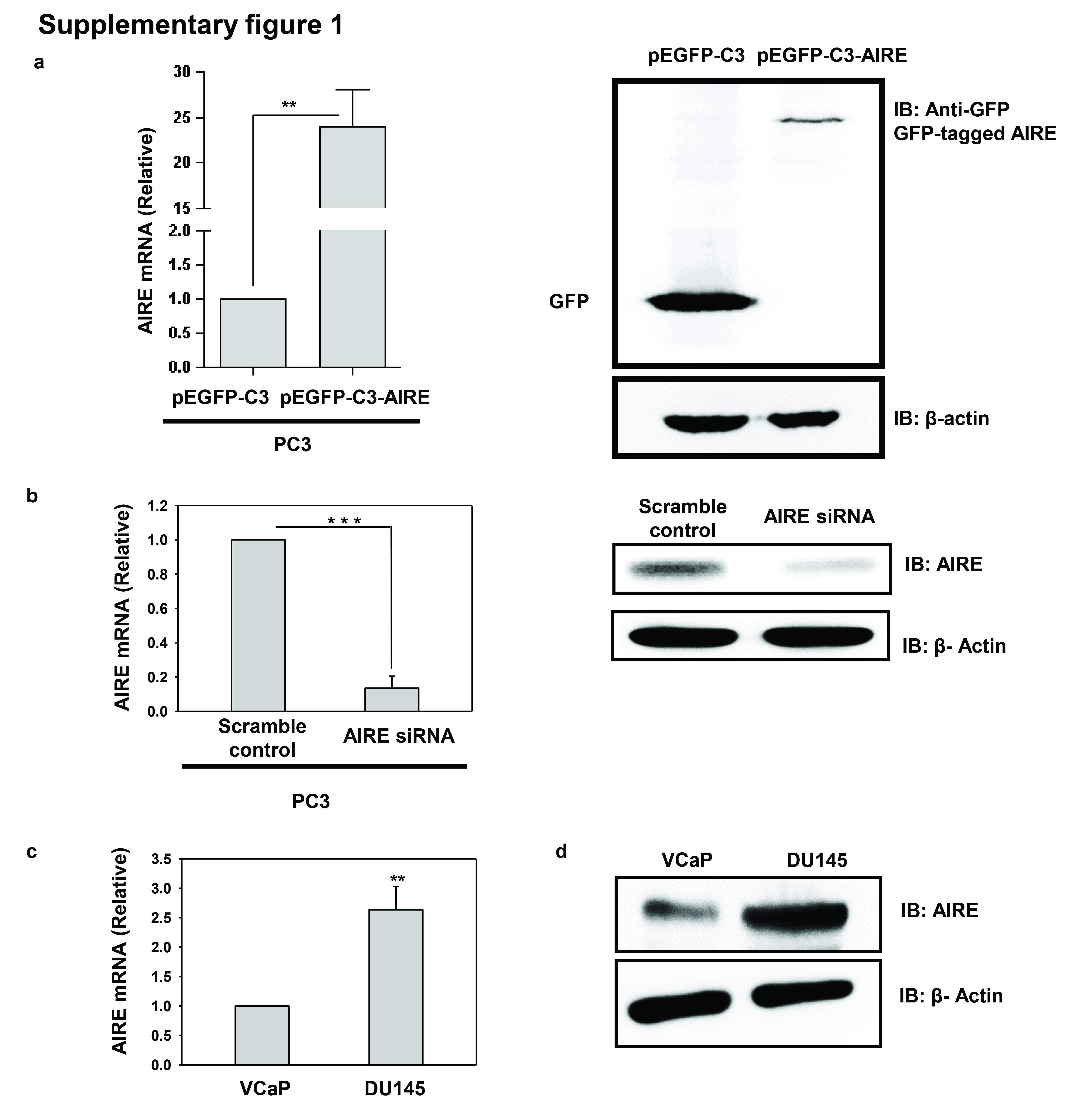

Supplement: Supplementary file 2 — Supplementary Figure 1 [file 41389_2018_53_MOESM2_ESM.tif]

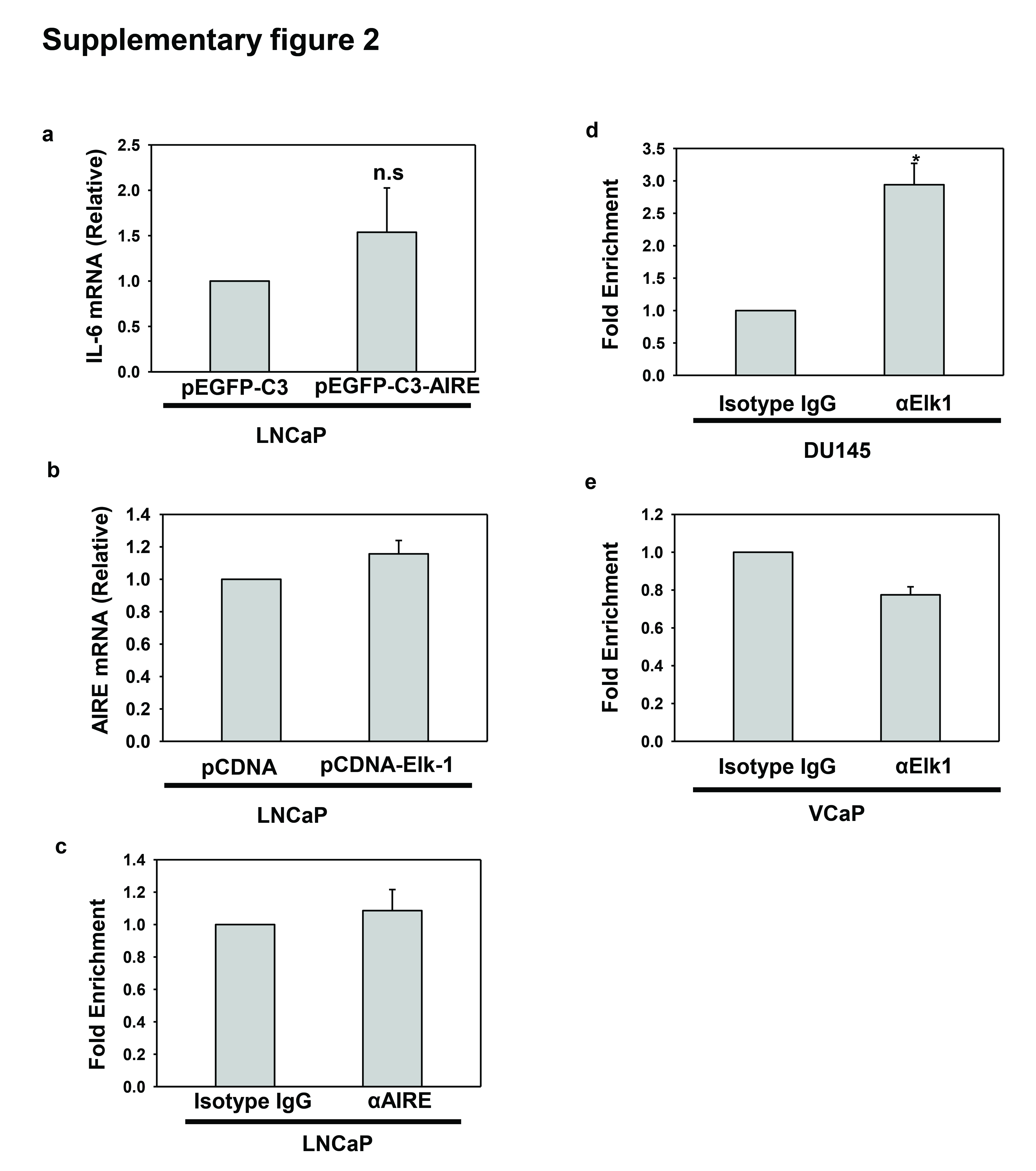

Supplement: Supplementary file 3 — Supplementary Figure 2 [file 41389_2018_53_MOESM3_ESM.tif]

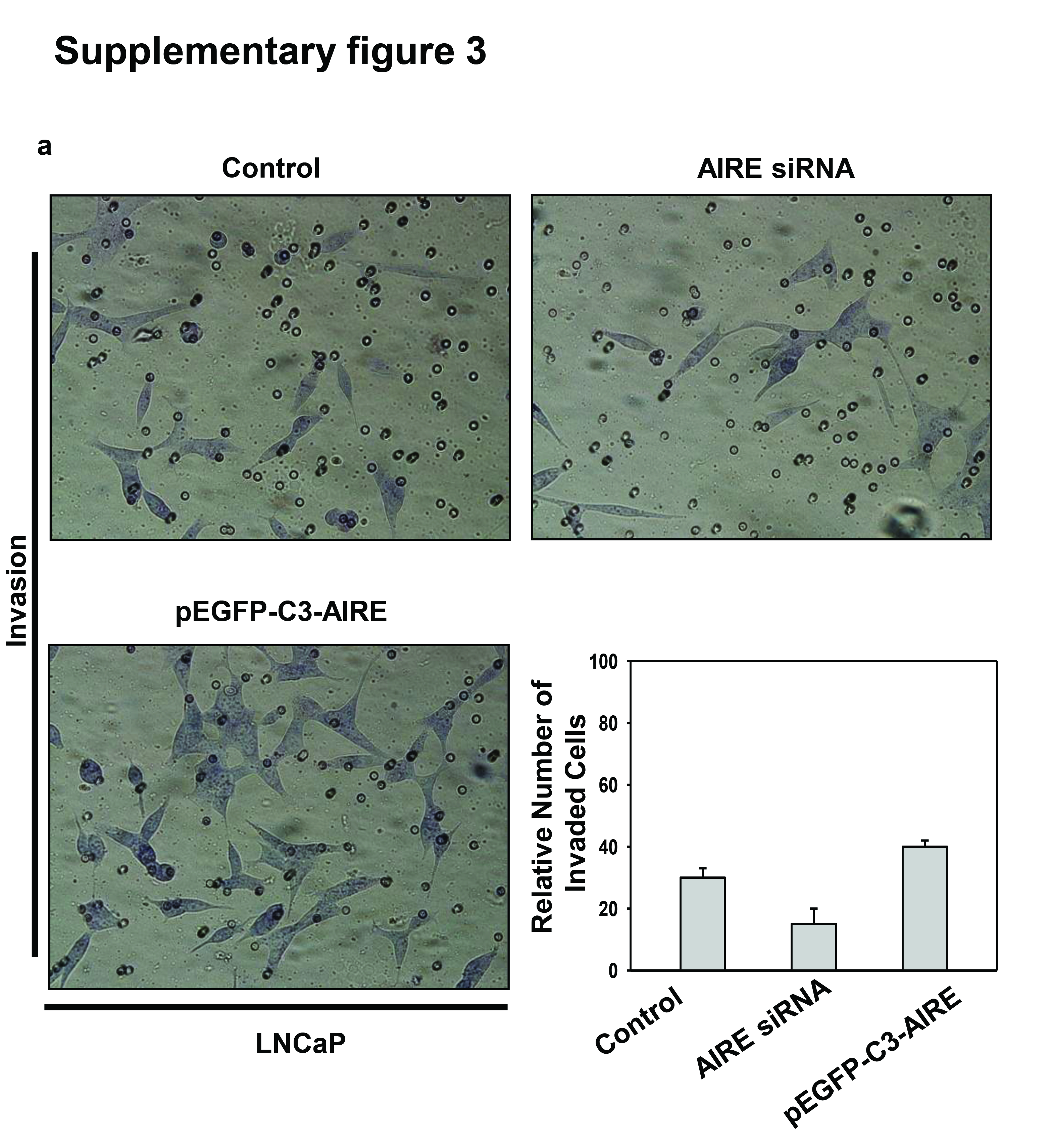

Supplement: Supplementary file 4 — Supplementary Figure 3 [file 41389_2018_53_MOESM4_ESM.tif]

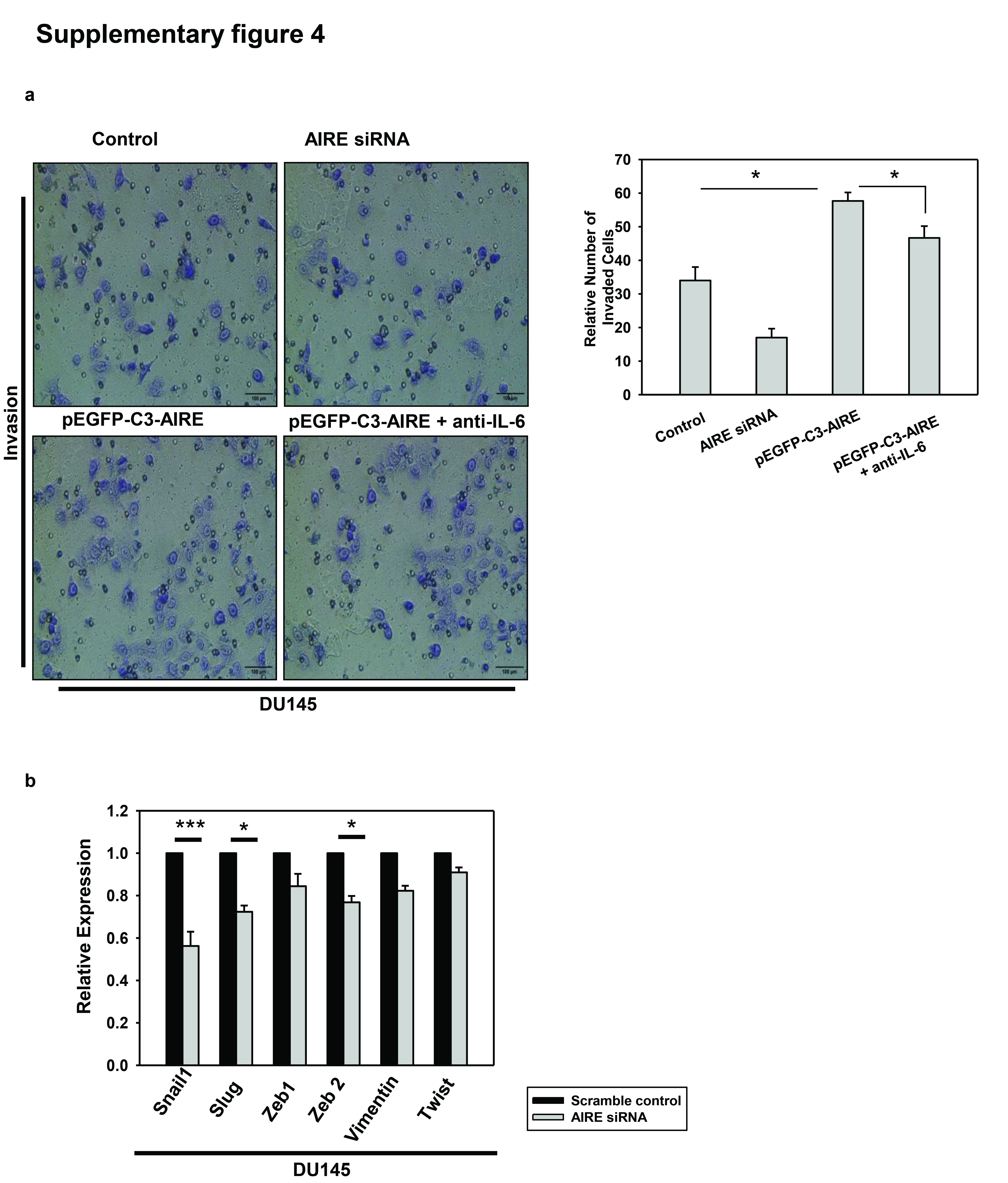

Supplement: Supplementary file 5 — Supplementary Figure 4 [file 41389_2018_53_MOESM5_ESM.tif]

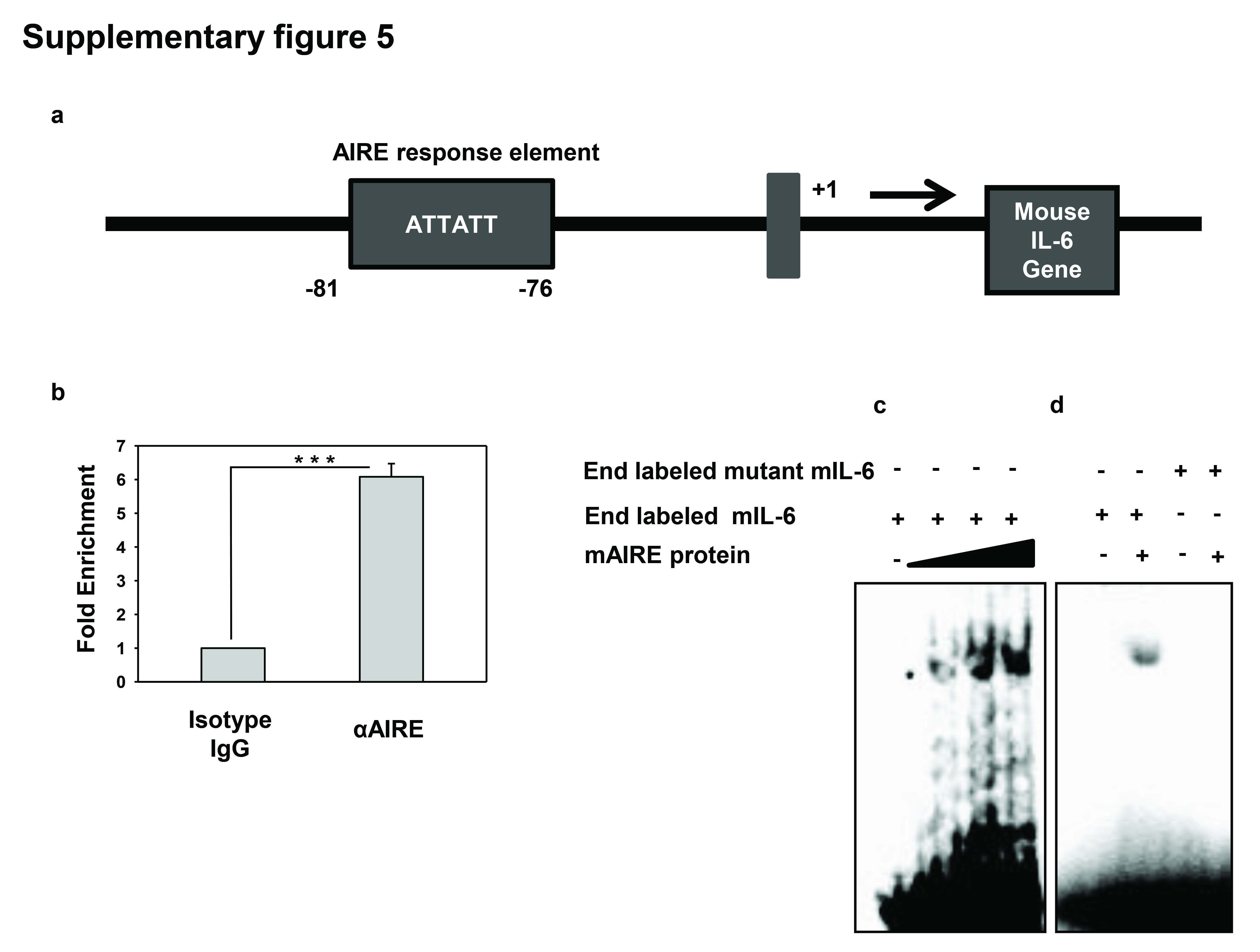

Supplement: Supplementary file 6 — Supplementary Figure 5 [file 41389_2018_53_MOESM6_ESM.tif]
